# Citrulline Accumulation Mechanism of *Pediococcus acidilactici* and *Weissella confusa* in Soy Sauce and the Effects of Phenolic Compound on Citrulline Accumulation

**DOI:** 10.3389/fmicb.2021.757542

**Published:** 2021-12-03

**Authors:** Kai Zhou, Xiao Zhang, Bingyong Li, Chaoqun Shen, Yuan-Ming Sun, Jianyuan Yang, Zhen-Lin Xu

**Affiliations:** ^1^Institute of Jiangxi Oil-Tea Camellia, Jiujiang University, Jiujiang, China; ^2^Guangdong Provincial Key Laboratory of Food Quality and Safety, College of Food Science, South China Agricultural University, Guangzhou, China; ^3^Department of Production-Learning-Research, Shenzhen Total-Test Technology Co., Ltd., Shenzhen, China

**Keywords:** ethyl carbamate, arginine deiminase pathway, citrulline, soy sauce, *Weissella confusa*, *Pediococcus acidilactici*

## Abstract

Citrulline is one of the major precursors of ethyl carbamate in soy sauce, and the accumulation of citrulline is attributed to the metabolism of arginine by bacteria with the arginine deiminase (ADI) pathway. However, key strains and factors affecting citrulline accumulation are not yet clear. In this study, two key strains of *Pediococcus acidilactici* and *Weissella confusa* were isolated from soy sauce *moromi*, and the regularity of citrulline formation was studied. Results showed that the conversion rates from arginine to citrulline (*A*/*C* rate) and the citrulline accumulation ability of *W. confusa* and *P. acidilactici* significantly increased in the presence of different concentrations of NaCl, indicating that salt stress was the main factor for citrulline accumulation. The inconsistent expression of *arc* genes by salt stress was the reason for citrulline accumulation for *P. acidilactici*, but for *W. confusa*, it may be due to the influence of arginine/citrulline on the transportation system: the intracellular citrulline could neither transport to extracellular space nor convert into ornithine. Environmental factors greatly influenced citrulline accumulation of the two key bacteria; *A*/*C* rate and citrulline formation in both strains decreased at low temperature (15°C) under high salt stress, but opposite effects were observed for the two key strains under anaerobic light condition. Moreover, quercetin and gallic acid significantly decreased the *A*/*C* rate and citrulline accumulation ability of the two key strains. The optimal quercetin and gallic acid as suggested by simulation experiment were 100 and 10 mg/l, respectively, and the lowest *A*/*C* rate of 28.4% and citrulline level of 1326.7 mg/l were achieved in the simulation system. This study explored the main factors for citrulline formation by the two key strains and proposed a targeted way to control citrulline in soy sauce.

## Introduction

Soy sauce, a traditional fermented condiment, is widely used for food preparation in Asian countries. Many consumers are attracted by its nutrition and good flavor, but soy sauce can also be contaminated by some harmful substances, such as ethyl carbamate (EC), a group 2A carcinogenic compound classified by the International Agency of Research on Cancer (IARC) since 2007 (Allen et al., 2010). It has been reported that even though the EC level in soy sauce is not as high as that in ethanol beverages, the estimated intakes of EC from soy sauce are still relatively high due to the high consumption of soy sauce in vulnerable subgroups and the relatively high EC content among fermented foods (Chen et al., 2015; Choi et al., 2017, 2018).

Citrulline and ethanol are considered to be the major EC precursors in soy sauce (Matsudo et al., 1993; Zhou K. et al., 2017; Zhou et al., 2019). However, reducing ethanol content may damage the quality of soy sauce, since a certain amount of ethanol has a major contribution to the flavor and spice taste of soy sauce. Therefore, mitigating EC content by reducing citrulline content is widely studied in recent years. Citrulline is mainly generated through the arginine deiminase (ADI) pathway, which is composed of three critical enzymes and amino acids. Arginine deiminase (encoded by *arcA*) and ornithine transcarbamylase (OTC, encoded by *arcB*) participate in the citrulline formation, and citrulline can be metabolized by OTC into carbamoyl-P and then utilized by carbamate kinase (CK, encoded by *arcC*) to produce CO_2_, ammonia, and ATP. The accumulation of citrulline is the result of the imbalance between the formation and elimination of citrulline (Jiao et al., 2014). In soy sauce, approximately 1 mg/ml citrulline will accumulate in the lactic acid fermentation stage, and it will then react with ethanol *via* ethanolysis reaction to produce EC during heating process (Matsudo et al., 1993; Zhang et al., 2015; Zhou et al., 2019). Apart from citrulline, high levels of arginine are also conducive to EC formation, but ornithine has the opposite effect during pasteurization (Zhou et al., 2021).

Citrulline is mainly formed in soy sauce during *moromi* fermentation, which involves complex microbial interactions. The dominant microflora in soy sauce, such as *Weissella*, *Staphylococcus*, and *Tetragenococcus*, are able to degrade arginine to citrulline *via* the ADI pathway (Knig et al., 2009; Tanaka et al., 2012; Fang et al., 2018). Therefore, key strains play a critical role in both citrulline formation and reduction. Reports have shown that *Pediococcus acidilactici* isolated from soy sauce *koji* (Zhang et al., 2015) and *Lactobacillus brevis* and *Lactobacillus buchneri* (Araque et al., 2011) isolated from wine and beer exhibited a high citrulline accumulation ability. Other factors, such as cellular energy level and environmental stress, including temperature, sugar concentration, pH, NaCl, and arginine concentration, can influence citrulline accumulation and soy sauce flavor by changing the conversion rate from arginine to citrulline (*A*/*C* rate) and arginine to ornithine (*A*/*O* rate). Zhang et al. (2015) and Fang et al. (2018) reported that a high concentration of NaCl and 2% ethanol content exhibited a significant effect on citrulline accumulation by affecting ADI pathway microbes. Environmental pH also showed a considerable impact on the citrulline formation pattern of *Lactobacillus fermentum* (Vrancken et al., 2009a).

Presently, reducing the biosynthesis of EC precursors is a general way to control EC in fermentation. Supplementation of strains with high arginine consumption ability and low citrulline accumulation capacity as a starter is a common method to reduce the citrulline and subsequent EC content (Zhang and Fang, 2016; Yu et al., 2020). However, fermented *moromi* is a complex system involving a variety of species that produce complex and special aroma, and it is difficult to reduce citrulline content by only changing the dominant lactic acid bacteria in the spontaneous fermentation. In recent years, phenolic compounds received considerable attention due to their potential antioxidant activity, and their effects on ameliorating EC-induced toxicity (Chen et al., 2016). Interestingly, phenolic compounds are able to regulate ADI enzyme in terms of reducing or increasing arginine consumption (Alberto et al., 2012). It has been reported that adding gallic or protocatechuic acid during yellow rice wine leavening can decrease the EC content by up to 91.9% (Zhou W. et al., 2017). Soy sauce is also rich in phenolic compounds contributed by the raw material of soybean and wheat (Peng et al., 2018). In this study, we screened two key strains of *P. acidilactici* and *Weissella confusa* from soy sauce *moromi* and clarified their citrulline metabolism regulation. Here, we also investigated the impact of phenolic compounds on the growth of key strains as well as their effect on citrulline accumulation, with the aim to explore its application potential in EC control in soy sauce.

## Materials and Methods

### Chemicals and Materials

Soy sauce *moromi* samples that have been fermented for 20–90 days (when citrulline was significantly increased) were collected from Guangdong Meiweixian Flavoring Foods Co., Ltd. L-citrulline, arginine, ornithine hydrochloride, and glucose were purchased from Shanghai Aladdin Reagent Co., Ltd. Yeast extract; peptone; and de Man, Rogosa and Sharpe (MRS) and nutrient broth (NB) medium were purchased from Guangdong Huankai Microbial Technology Co., Ltd. Bacterial genomic DNA extraction kit, RNAsimple total RNA extraction kit, and FastKing-RT SuperMix kit were purchased from Tiangen Biochemical Technology (Beijing) Co., Ltd. Real-Time PCR Super Mix (SYBR Green with anti-Taq) kit was purchased from Beijing Polymer Biotechnology Co., Ltd.

### Key Strain Screening

The screening of citrulline accumulation microbes was performed using the methods of Zhang et al. (2015) with some modifications. Firstly, the *moromi* samples were serially diluted with 0.9% NaCl (*w*/*v*), plated on MRS and NB agar supplemented with 80 mg/l cycloheximide and 2% NaCl, and then stationary cultured at 30°C for 2–5 days. Subsequently, single colonies were subcultured on MRS and NB agar supplemented with 4 g/l arginine and 0.005% bromocresol purple (*w*/*v*) to test the arginine utilization ability. The strains able to utilize arginine were distinguished by forming a purple halo around the colonies. Finally, the candidates were inoculated in MRS (or NB) broth with 18% NaCl (*w*/*v*) and 10 g/l arginine and the modified soy sauce broth with 10 g/l arginine, then cultured at 30°C for 7 days. The arginine and citrulline content were determined, and the *A*/*C* rate and *A*/*O* rate were calculated by Eq. 1.


(1) $$\mathrm{Formula1}:\mathrm{rate}\left(\%\right)=\frac{\left(\frac{{\text{C}}_{1}\times \text{V}}{{\text{M}}_{1}}\right)}{\left(\frac{{\text{C}}_{1}\times \text{V}}{{\text{M}}_{1}}\right)}*100$$

where *C*_1_ is the citrulline (or ornithine) content, *M*_1_ is the molar mass of citrulline (or ornithine), *C*_2_ is the arginine consumption, *M*_2_ is the molar mass of arginine, and *V* is the volume.

### Growth Conditions and Fermentation Experiment

Isolates (*W. confusa* and *P. acidilactici*) were grown in 50 ml MRS broth at 30°C and statically cultured to the late-log phase, as assessed by OD600; the cells were harvested by centrifugation at 8,000 × *g* for 4 min at 4°C, then washed twice with 0.9% NaCl. The cell pellets were resuspended in saline with OD600 ≈ 1, then 1 ml of strain solution was cultured in 9 ml of modified MRS broth for static culture. The modified MRS broth were based on a medium [peptone (4 g/l), meat extract (5 g/l), yeast extract (5 g/l), sodium acetate (5 g/l), K_2_HPO_4_ (2 g/l), triammonium citrate (2 g/l), MgSO_4_⋅7H_2_O (0.2 g/l), MnSO_4_⋅4H_2_O (0.05 g/l), and Tween 80 (1 g/l)] with addition of NaCl [6, 9, 12, 15, or 18% (*w*/*v*)], arginine (2, 4, or 6 g/l), citrulline (2 or 4 g/l), ornithine (0.5 or 1 g/l), glucose (0, 20, 40, or 80 g/l), urea (5, 10, 20, or 40 g/l), ethanol [0.5, 1, 2, or 4% (*w*/*v*)], and glycerin [0.5, 1, 2, or 4% (*w*/*v*)]. The pH of the medium was adjusted to 4, 4.8, 5.6, 6.4, or 7.2 by acetic acid/NaOH, and the incubation temperatures were set at 15°C, 30°C, and 37°C, respectively. Except for the groups added with NaCl that were cultured at 30°C for 48 h, other groups were cultured for 24 h. After being cultured, samples were centrifuged at 12,000 × *g* for 6 min, and the supernatants were taken to measure the citrulline and arginine contents. For gene expression and intercellular and extracellular amino acid analysis, the cells were cultured for 5 h.

### Phenolic Compounds

The phenolic compounds were dissolved in ethanol at the concentration of 10 mg/ml, then filtered through a membrane filter for sterility. The modified MRS fortified with 10 g/l arginine and 18% NaCl was sterilized and cooled down to room temperature. Then, the phenolic solution was added into the sterilized medium to obtain a final concentration of 100 mg/l. The control sample was added with the same volume of ethanol. The prepared cell pellets were then resuspended in the above culture for static culture at 30°C. Arginine, citrulline, and ornithine contents of supernatants were subsequently analyzed.

### Simulation Experiment

The fermented soy sauce *moromi* is a complex system involving a variety of species, and a central composite design (CCD) experiment was applied to verity the effect of main factors and to optimize the phenolic compounds on citrulline accumulation (Supplementary Table 1). The fresh *moromi* (after 20 days of fermentation) fortified with 4 mg/l arginine was treated as the simulated fermentation sample. The arginine consumption, the formation of citrulline and ornithine, the *A*/*C* rate, and the *A*/*O* rate were considered as dependent variables. The experimental design and data analysis were performed using the Design Expert software, version 8.0.6.

### Analytical Methods

#### Intercellular and Extracellular Amino Acid Detection

The cultured strains were centrifuged at 12,000 × *g* for 6 min, and the precipitate and supernatant were collected for intercellular and extracellular amino acid detection, respectively. An equal volume of 4% sulfosalicylic acid was added to the supernatant and fully mixed then stood for 30 min at 4°C; the mixture was centrifuged at 12,000 × *g* for 10 min, and the supernatant was collected for extracellular arginine, citrulline, and ornithine determination. The precipitate was washed twice with 0.9% NaCl, resuspended in 2 ml cold 0.9% NaCl solution, and crushed by an ultrasonic crusher (Scientz-IID, SCIENTZ, China). Cell debris were removed by centrifugation, then 4% sulfosalicylic acid was used for protein precipitation. Supernatant was collected for intercellular arginine, citrulline, and ornithine determination. The determination of arginine, citrulline, and ornithine contents was based on a previous description (Arena et al., 1999; Zhou W. et al., 2017).

#### Arginine Deiminase-Related Gene Expression

The strains from the third subculture were harvested at the end of the logarithmic growth phase and then cultured in a specified medium for 5 h. The total RNA was extracted using the RNAsimple total RNA extraction kit and reverse transcribed with FastKing-RT SuperMix kit, according to the manufacturer’s instruction. RT-qPCR for analysis of relative *arc* gene expression was performed using a Real-Time PCR Super Mix and a Rotor-Gene 6000 Real-Time PCR System (QIAGEN, Australia). The relative expression levels of the *arc* operon genes at different conditions or cultures were calculated based on a standard curve. Primers used are listed in Supplementary Table 2.

#### Enzyme Activity Evaluation

The cultivated strains were collected by centrifugation and washed twice in 2 ml of cold 0.9% NaCl solution. The cells were then resuspended at 2.5% (*w*/*v*) in 0.2 M sodium phosphate buffer (pH 6.5) for determination of ADI activity and in 0.2 M sodium acetate buffer (pH 5.8) for determination of OTC activity. The suspension cells were crushed by an ultrasonic crusher (Scientz-IID, SCIENTZ, China), and enzyme activities were determined according to a previous description (Spano et al., 2007). The enzyme activity (*U*) was defined as the amount of substrate (μmol) (arginine and citrulline for ADI and OTC, respectively) consumed per minute and per microgram of protein.

### Statistical Analysis

All experiments were performed in triplicate unless otherwise noted, with the mean value and standard error reported. The data were analyzed by ANOVA using SPSS (version 22, IBM, United States), and significant differences among the mean values were determined by adopting Tukey’s multiple range test, with significance defined at *p* < 0.05.

## Results

### Screening of Key Strain for Citrulline Accumulation From Moromi

A total of 34 strains were isolated from *moromi*, and all strains exhibited the ability to degrade arginine and accumulated variable concentrations of citrulline besides *Leuconostoc garlicum*. We found that six strains were detected to possess a strong ability to decompose arginine and accumulate citrulline through screening tests. Among these isolates, two strains, which were identified as *P. acidilactici* and *W. confusa* (the sequence of 16S DNA is shown in the Supplementary Material), exhibited an outstanding citrulline accumulation capability (Table 1). The *A*/*C* rates *P. acidilactici* and *W. confusa* reached up to 40.9% and 30.2%, respectively, and citrulline was accumulated over 900 mg/l. Representative strains of *P. acidilactici* and *W. confusa* isolated from *moromi* were selected for further analysis.

**TABLE 1:** The arginine consumption and citrulline accumulation ability of crucial bacteria in modified MRS broth.

Strain	Arginine (mg/l)	Citrulline (mg/l)	*A*/*C* rate (%)
*Weissella confusa*-5	717.3 ± 46.1	998.2 ± 70.7	30.2
*Weissella* sp.-4	728.2 ± 16.4	424.5 ± 17.0	12.9
*Pediococcus acidilactici*-1	970 ± 13.5	1,247 ± 128.4	40.9
*Leuconostoc* sp.-1	1,219 ± 40.7	525.7 ± 17.0	18.8
*Sphingomonas echinoides*-2	878.2 ± 38.6	429.0 ± 12.8	13.7
*Rummeliibacillus stabekisii*-1	1,007.4 ± 33.0	627.0 ± 46.2	20.8

### The Effect of Medium Constituent Condition on Arginine Metabolism

**FIGURE 1:**
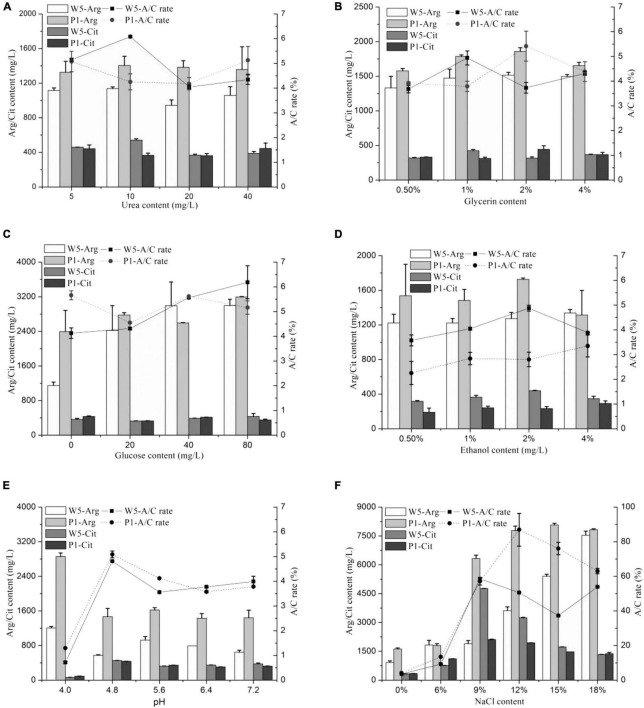
The effect of medium condition [**(A)** urea, **(B)** glycerin, **(C)** glucose, **(D)** ethanol, **(E)** pH, and **(F)** NaCl] on citrulline accumulation of *W. confusa* and *P. acidilactici* (W5, *W. confusa*; P1, *P. acidilactici*; Arg, arginine; Cit, citrulline; similarly hereinafter).

The effects of different medium constituent conditions (urea, glucose, ethanol, glycerol, NaCl, and pH) on citrulline accumulation of the two strains are shown in Figure 1. The *A*/*C* rate was approximately 6% in the mediums containing different concentrations of urea, glucose, ethanol, and glycerol cultured with *W. confusa* and *P. acidilactici*, which was much lower than that cultured in modified MRS broth (above 30%). Moreover, those constituents had little influence on citrulline accumulation, directly indicating a small effect on citrulline accumulation. The *A*/*C* rates were even less than 2% when *W. confusa* and *P. acidilactici* were cultured at a medium with pH 4.0, and the growth rate of the strains was inhibited severely; a similar trend was found in the arginine catabolism. These stains were isolated from *moromi* when the pH was 4.8, which was the optimal pH for citrulline accumulation.

The addition of different contents of salt significantly affected the citrulline accumulation capacity of *W. confusa* and *P. acidilactici*. These strains had a strong ability to consume arginine at salinity less than 6%, and citrulline was less than 1,000 mg/l. The arginine utilization capacity rapidly decreased when the NaCl concentration was higher than 9%. When NaCl was at 9%, the highest citrulline concentration was found for both strains, and the *A*/*C* rates significantly increased to approximately 60%. When NaCl increased to 18%, both stains exhibited a strong citrulline accumulation capacity in comparison with the control group, and the A/C rate and citrulline content were slightly higher in *P. acidilactici* than those in *W. confusa*. Then, we cultured the strains in modified *moromi* culture, and the results showed that the *A*/*C* rates of *W. confusa* and *P. acidilactici* were 51.3% and 44.0%, respectively (Supplementary Table 3). These evidences confirm that the NaCl concentration plays a key role in citrulline accumulation in soy sauce.

**FIGURE 2:**
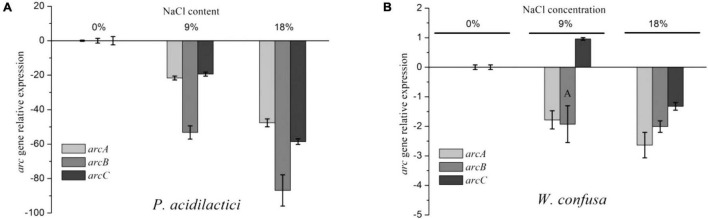
*arc* gene relative expression of *P. acidilactici* **(A)** and *W. confusa* **(B)** in the presence of different NaCl contents.

The ADI pathway gene expression of *W. confusa* and *P. acidilactici* was determined under different NaCl concentrations (0, 9, and 18%). As shown in Figure 2, the expression of *arcA* and *arcC* genes was significantly repressed in *P. acidilactici* by NaCl, decreasing by approximately 20-fold and 50-fold at 9 and 18% NaCl, respectively. The expression of the gene *arcB* showed a higher reduction at the same conditions, which were, respectively, 53-fold and 86-fold for the two strains. These results mean that the expression of *arcB* was relatively lower than that of *arcA* and *arcC* in the presence of NaCl, especially at 9% NaCl. Likewise, the enzyme activity of the ADI pathway was significantly decreased in the presence of NaCl, and OTC activity declined even more (Supplementary Figure 1). Therefore, the unequal proportional inhibition of ADI and OTC activity directly led to the reduction of arginine utilization and the increase of citrulline accumulation. However, the reduction of ADI and OTC by NaCl was lower than that of relative gene expression.

**FIGURE 3:**
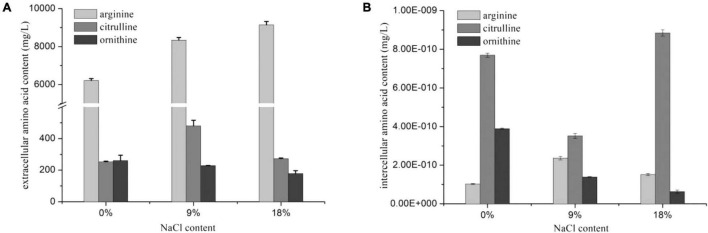
Extracellular **(A)** and intercellular **(B)** amino acids of *W. confusa* in the presence of different NaCl contents.

The *arc* gene expression of *W. confusa* was found to be different with *P. acidilactici*. The expression of *arcA* and *arcB* genes in *W. confusa* slightly decreased (by approximately two-fold) in the presence of NaCl, and the expression rate of *arcA/arcB* was about 1.0, indicating that citrulline accumulation in *W. confusa* was not caused by the unequal down-regulation of *arc* genes. In order to further investigate the citrulline accumulation mechanism of *W. confusa*, the intercellular and extracellular amino acids were determined at different NaCl concentrations for 5 h. As shown in Figure 3, the sum of citrulline and ornithine content was less than 70% of arginine consumption, especially without NaCl, which indicates that the ADI pathway was not the only way for arginine consumption. Furthermore, arginine metabolism was very different in the presence of NaCl: (1) ornithine was the main product of the ADI pathway when cultivated without NaCl, (2) when cultivated at 9% NaCl, citrulline in the extracellular space was largely increased and ornithine was decreased, and (3) when the NaCl concentration was increased to 18%, the growth of *W. confusa* was significantly inhibited, so did the arginine and citrulline transportation ability, leading to the accumulation of citrulline inside the cells. These results indicate that the different arginine/citrulline transportation abilities under different salt stresses may be the reason for the citrulline accumulation in *W. confusa*.

**FIGURE 4:**
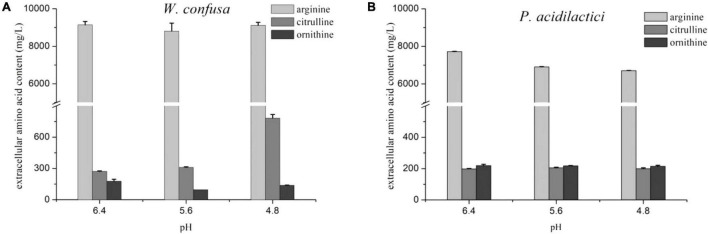
The effect of pH on arginine metabolism of *W. confusa* **(A)** and *P. acidilactici* **(B)** in the presence of 18% NaCl.

The citrulline content and *A*/*C* rate of the two strains were slightly increased at pH 4.8. The effect of pH on citrulline accumulation was then investigated in the presence of 18% NaCl. As shown in Figure 4, *P. acidilactici* possessed a stronger arginine consumption ability than *W. confusa* in the medium with NaCl. Citrulline accumulation in *P. acidilactici* remained stable at three pH values at 18% NaCl, while it exhibited a significantly higher citrulline accumulation capacity at pH 4.8 than that at pH 5.6 and 6.4 in *W. confusa*. The results indicate that pH can regulate arginine metabolism in the presence of high concentrations of NaCl. There was no significant change in *arcA* and *arcB* gene expressions in *W. confusa* under different pH conditions in 18% NaCl, but a general over-expression (six–seven-fold) of *arcC* was observed at a low-pH condition (Supplementary Figure 2). The low intercellular arginine and citrulline content at pH 4.8 indicates that large amounts of arginine were converted to citrulline and then secreted into the medium.

### The Effect of Cultivation Environment on Arginine Metabolism and Citrulline Accumulation

**FIGURE 5:**
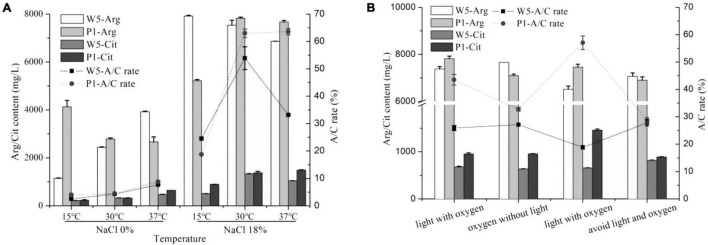
The effect of temperature **(A)** and light and oxygen **(B)** on citrulline accumulated by *W. confusa* and *P. acidilactici.*

Our previous study showed a significant reduction of citrulline when the temperature at the primary fermentation period in soy sauce was set at 15°C (Zhou et al., 2019). In this study, the arginine consumption in *P. acidilactici* was lesser at 15°C than that at 30 and 37°C without the addition of NaCl, whereas the opposite behavior was observed in *W. confusa*. Both strains accumulated minimal citrulline at 15°C (Figure 5). As previously mentioned, the *A*/*C* rates did not exceed 10% at any culture temperature without NaCl, while they were significantly increased at 18% NaCl, especially when the strains were cultured at 30°C, the *A*/*C* rate improved by 10–15-fold. Noticeably, both strains exhibited the lowest *A*/*C* rate (lower than 25%) and citrulline content at 15°C, which was much lower than that at 30°C and 37°C, directly explaining the reason of low accumulation of citrulline at low fermentation temperature in soy sauce. Under 18% NaCl, the *arc* gene expression changes of the two strains were below two-fold at different temperatures (Supplementary Figure 3), suggesting the small effect of temperature on the ADI pathway under high salt stress.

In low-salt solid-state fermented soy sauce, several times of “daochi” during the fermentation process can increase the uniformity and oxygen content of the *moromi*. While for traditional Chinese-type soy sauces, the *moromi* is usually fermented in open air for almost 6 months. The effects of oxygen and light on citrulline accumulation of the key strains were then investigated. Small differences of *A*/*C* rate and citrulline accumulation were observed in *W. confusa* when cultured at four environmental combinations of light and oxygen. However, in *P. acidilactici*, the light improved citrulline accumulation, and the maximum citrulline content (1,459.0 mg/L) and A/C rate (57.1%) were observed in light and anaerobic condition. The opposite results were observed in *W. confusa*.

### The Effect of Phenolic Compounds on Arginine Metabolism and Citrulline Accumulation

**FIGURE 6:**
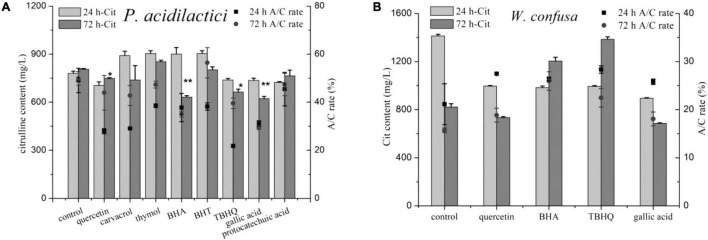
The effect of phenolic compounds on citrulline accumulation of *P. acidilactici* **(A)** and *W. confusa* **(B)**. * and ** means the significant difference (*P* < 0.05) and most significant difference (*P* < 0.01) in 72 h-Cit compared with control.

Eight phenols were added into the medium to test their effects on citrulline accumulation of the two strains, and both positive and negative effects were observed (Figure 6). In the control group, the *A*/*C* rate and citrulline in *P. acidilactici* at 24 h were 780.38 mg/l and 49.04%, respectively, and increased slightly after 72 h of culture. Noticeably, nearly all of the tested phenols exhibited a strong inhibitory effect against the *A*/*C* rate of *P. acidilactici* at 24 h, but the *A*/*C* rate then increased after 72 h of culture. Quercetin, TBHQ, gallic acid, and protocatechuic acid showed an inhibitory effect on citrulline formation by *P. acidilactici* cultured for 24 h. After 72 h of culture, the level of citrulline in the protocatechuic acid group was increased and had no significant difference compared with the control group, but the citrulline content in the BHA group dropped by nearly half. However, the phenols that showed citrulline inhibitory effects on *P. acidilactici* exhibited the opposite effect on *W. confuse*; they rapidly improved the *A*/*C* rates, and citrulline accumulation in BHA and TBHQ was significantly increased after 72 h of culture. In summary, among the tested phenols, only quercetin and gallic acid with 100 mg/l could be the potential inhibitors of citrulline formation in soy sauce, and they also showed an inhibitory effect on microbial growth (Supplementary Figure 4).

Fermented *moromi* is a complex system. In order to verify the actual inhibitory effect of quercetin and gallic acid on *moromi*, a CCD experiment was designed with two major factors (pH and temperature). Arginine, citrulline, and ornithine content was determined after 48 h and 7 days of culture, and arginine consumption, citrulline and ornithine formation, *A*/*C* rate, and *A*/*O* rate were set as the responses. The validity of the models was statistically analyzed by ANOVA as presented in Table 2. The desirable model with *p*-values < 0.05 and lack of fit >0.05 indicated that the model terms were significant while the pure error was not significant. We found the models of 7 days-Arg, 48 h and 7 days-ornithine, and 7 days-A/C and A/O rate could well fit the real data. The 20-day fermented *moromi* supplied with 4 mg/ml arginine was used as a simulated fermented model; therefore, citrulline was not only accumulated by *P. acidilactici* and *W. confusa*. Among those models, only temperature and quercetin show a significant linear effect (*p* < 0.05) on all parameters and the 7 days-A/C rate model, while pH and gallic acid were less important. According to these two models, we set the pH value as original value and the temperature at 15°C. The optimum quercetin and gallic acid contents as suggested by the software were 100 and 10 mg/l, respectively, corresponding to the lowest *A*/*C* rate of 29.8% and the highest *A*/*O* rate of 34.1%. To verify the prediction, cultured *moromi* in the presence or absence of quercetin and gallic acid was analyzed in triplicate to determine the optimal A/C and A/O rates. The A/C rate and A/O rate obtained at these conditions are 28.4 and 33.9%, respectively, corresponding to citrulline of 1,326.7 mg/l, which was 17.3% lower than that of *moromi* without phenols (Table 3).

**TABLE 2:** ANOVA for response surface quadratic model for CCD experiment (*P**-***value).

RV	Model	A	B	C	D	AB	AC	AD	BC	BD	CD	Lack of fit
48 h-Arg	0.76	0.99	0.11	0.54	0.66	0.64	0.86	0.84	0.68	0.75	0.13	0.97
**7 days-Arg**	**0.04**	**0.48**	**<0.01**	**0.67**	**0.49**	**0.69**	**0.74**	**0.12**	**0.86**	**0.63**	**0.41**	**0.41**
48 h-Cit	0.16	0.45	0.02	0.93	0.32	0.37	0.9	0.88	0.11	0.06	0.88	0.82
7 days-Cit	<0.01	0.46	<0.01	0.33	0.77	0.22	0.71	0.87	0.78	0.73	0.90	<0.001
**48 h-Orn**	**<0.01**	**0.91**	**<0.01**	**0.15**	**0.06**	**0.08**	**0.45**	**0.08**	**0.93**	**0.88**	**0.93**	**0.97**
**7 days-Orn**	**<0.01**	**0.21**	**<0.01**	**0.70**	**0.18**	**0.68**	**0.11**	**0.04**	**0.27**	**0.75**	**0.60**	**0.43**
48 h-*A*/*C* rate	0.47	0.64	0.40	0.74	0.24	0.33	0.95	0.62	0.09	0.07	0.32	0.56
**7 days-*A*/*C* rate**	**<0.01**	**0.56**	**<0.01**	**0.04**	**0.91**	**0.08**	**0.84**	**0.39**	**0.52**	**0.86**	**0.67**	**0.06**
48 h-*A*/*O* rate	0.63	0.95	0.29	0.99	0.24	0.27	0.67	0.60	0.79	0.71	0.18	0.98
**7 days-*A*/*O* rate**	**<0.01**	**0.08**	**<0.01**	**0.56**	**0.38**	**0.89**	**0.19**	**<0.01**	**0.25**	**0.47**	**0.23**	**0.75**

*RV, response value; A, pH; B, temperature; C, quercetin; D, gallic acid; Arg, arginine consumption; Cit, citrulline formation; Orn, ornithine formation. The bold values means that the five model could well fit the real data.*

**TABLE 3:** The relationship between predicted value and actual value of simulated *moromi* (*n* = 3).

	Citrulline (mg/l)	Ornithine (mg/l)	*A*/*C* rate (%)	*A*/*O* rate (%)
Predicted value (7 days)	–*	400.1	29.8	34.1
Actual value (the optimal condition)	1,326.7 ± 43.9	414.5 ± 9.9	28.4	33.9
Actual value (absence of phenols)	1,603.8 ± 30.3	386.3 ± 20.3	34.5	28.4

*Unavailable model.

## Discussion

### Key Strains for Citrulline Accumulation

Citrulline has proven to be the major EC precursor in soy sauce, which mainly originates from the catabolism of arginine. Although *Saccharomyces cerevisiae* can produce citrulline *via* arginine metabolism, the generation of citrulline is majorly attributed to the metabolism of arginine by malolactic bacteria *via* the ADI pathway (Fang et al., 2018). In previous work, citrulline content in *moromi* was found to continuously increase throughout the fermentation period, and it was significantly accumulated when fermented from 20 to 90 days in Cantonese soy sauce (Zhou et al., 2019). In this study, we isolated 34 strains from *moromi* and determined their arginine utilization ability. The results showed that all strains exhibited the ability to degrade arginine and accumulate variable concentrations of citrulline except for *L. garlicum*. During *koji*-making, only six isolates were identified to be able to accumulate citrulline *via* the ADI pathway (Zhang et al., 2015). We speculated that citrulline did not accumulate in the *koji*-making period due to the low arginine content, low abundance of strain with ADI pathway, or lack of stimuli (NaCl). *P. acidilactici* and *W. confusa* were isolated from *moromi* as the key strains for citrulline accumulation in soy sauce. *Enterococcus faecium* and *Enterobacter* sp. showed strong arginine consumption and weak citrulline accumulation ability when cultured in the 18% NaCl medium, revealing that they can be potential candidates to reduce EC in soy sauce. Noticeably, one strain of *P. acidilactici* and five isolates of *W. confusa* were screened out from *moromi*, while different strains of *W. confusa* had different citrulline accumulation abilities in the presence or absence of NaCl. The available genome sequencing research revealed an unexpected diversity of both gene arrangement and composition of the ADI gene clusters. Even for closely related microorganisms, the ADI-related genes were organized in one or more clusters (Zúñiga et al., 2002). Further research is required to verify the difference of those closely related microorganisms’ ADI clusters and their effects on arginine metabolism. Beyond that, the ADI pathway was easily induced by substrate, cell energy level, and environmental factors (Araque et al., 2013; Zhou W. et al., 2017; Majsnerowska et al., 2018).

### Crucial Influence Factors for Citrulline Accumulation

The species of lactic acid bacteria, such as *Lactobacillus*, isolated from the winemaking process was mainly involved in malolactic fermentation. The ADI pathway genes and arginine degradation were deeply studied (Vrancken et al., 2009a; Araque et al., 2011, 2013; Rimaux et al., 2012). The effect of environmental pH and ethanol on arginine metabolism and citrulline release was determined by regulating the expression of ADI pathway genes. In case of *P. acidilactici* and *W. confusa*, the arginine metabolism was slightly affected by pH, ethanol, glycerin, glucose, and urea, while NaCl had a noticeable effect on citrulline accumulation. The *A*/*C* rate significantly increased in both strains when cultured under salt stress. Vrancken et al. (2009b) reported that the *A*/*C* rate of *L. fermentum* was not affected by temperature, but it increased with increasing NaCl concentrations, and salt directly facilitated the direct ornithine production. Zhang et al. (2015) pointed out that the presence of a high level of NaCl was a critical environmental factor for citrulline accumulation for *P. acidilactici* isolated from *koji*, and they advised that a better control of the production process to keep a low NaCl concentration may be a good strategy to reduce the risk of the product. However, in this study, *P. acidilactici* and *W. confusa* were found to accumulate more citrulline in the presence of 9–15% NaCl, and the *A*/*C* rate of *P. acidilactici* was higher at 9 and 15% NaCl than that at 18%. Therefore, fermentation at a low NaCl concentration may not only fail to reduce citrulline but also improve the citrulline accumulation. To the best of our knowledge, research on the regulation of the ADI pathway by salt stress is rare. We found that the expression of the three *arc* genes was suppressed in the presence of 18% NaCl, and the inconsistent reduction rate of *arcA*/*arcB* expressed in the ADI pathway was the reason causing the accumulation of citrulline in *P. acidilactici*, while the interference of the transport system of three key amino acids in the presence of NaCl was the reason for citrulline accumulation in *W. confusa*.

Mitigation of EC by lowering the temperature was proven to be an efficient method *via* reducing the reaction of EC precursors. Although the adaptability of *L. fermentum* through the ADI pathway under salt stress was much better than that under constant temperatures (Vrancken et al., 2009b), temperature also regulated the ADI pathway even in the presence of a high concentration of NaCl (Figure 5). For *P. acidilactici* and *W. confusa*, the *A*/*C* rate reduced to approximately 20% at 15°C, and citrulline content at 15°C was less than half of that at 30°C. In the absence of NaCl, the extracellular citrulline concentration in *L. fermentum* decreased with increasing temperature, while the ornithine concentration rose even higher (Vrancken et al., 2009b). However, a drastic reduction of the growth rate of *P. acidilactici* and *W. confusa* was observed at a high concentration of NaCl; actually, they stopped growing when the NaCl content in the culture medium was greater than 9%. The cells started to metabolize arginine mainly into citrulline and minor amounts of ornithine. Therefore, an alternative to reduce citrulline content is to lower the fermentation temperature.

### The Potential Citrulline Reduction Method for Soy Sauce Fermentation

Phenols have been proven to be effective to adjust the metabolism of arginine *via* the ADI pathway (Alberto et al., 2012). Gallic and protocatechuic acids can regulate EC catabolism and reduce the EC content by up to 91.9% in the course of yellow rice wine leavening by reducing the arginine consumption in *Lactobacillus hilgardii*, but the remaining citrulline concentration increased (Zhou W. et al., 2017). Similar effects were found for bamboo leaves extract (Zhou et al., 2020). Therefore, the accurate screening of target phenols is essential for the usage of phenolic compounds to decrease EC content in fermented food within a given environment. In this study, both positive and negative effects on citrulline accumulation in key strains were observed for different phenols, and quercetin and gallic acid were observed to be more efficient to inhibit citrulline accumulation. Furthermore, the simulation experiment results indicated that quercetin had a significant effect on the *A*/*C* rate in the fermentation process of *moromi*, and the *A*/*C* rate in fermented liquor with optimum phenols was 17.7% lower than that in the control group. In addition, gallic acid did not significantly affect EC formation, but quercetin significantly reduced EC content by 48.38% by preventing the transformation from citrulline to EC (Zhou et al., 2021). The addition of quercetin and gallic acid improved the *A*/*O* rate by up to 33.9%, resulting in high ornithine residues, which was also beneficial for the reduction of EC (Fang et al., 2013; Zhou et al., 2021). Quercetin and gallic acid were the common phenolic compounds in fermented food with strong antioxidant and anticancer activity (Sachithanandam et al., 2020). Therefore, future study may focus on investigating the exact inhibiting effect and mechanism of quercetin and gallic acid on citrulline accumulation and EC formation.

This study provides a better understanding on the citrulline accumulation mechanism by the key strains in soy sauce and also proposes a potential method for effectively controlling citrulline by lowering fermentation temperature and addition of polyphenols.

## Data Availability Statement

The original contributions presented in the study are included in the article/Supplementary Material, further inquiries can be directed to the corresponding author/s.

## Author Contributions

KZ, Y-MS, and Z-LX conceived and designed the project. KZ, XZ, BL, and CS performed the experiments. KZ and JY analyzed the data. KZ and Z-LX wrote the manuscript. All authors contributed to the article and approved the submitted version.

## Conflict of Interest

KZ and CS were employed by Shenzhen Total-Test Technology Co., Ltd. The remaining authors declare that the research was conducted in the absence of any commercial or financial relationships that could be construed as a potential conflict of interest.

## Publisher’s Note

All claims expressed in this article are solely those of the authors and do not necessarily represent those of their affiliated organizations, or those of the publisher, the editors and the reviewers. Any product that may be evaluated in this article, or claim that may be made by its manufacturer, is not guaranteed or endorsed by the publisher.
