# Supplementary material for: Citrulline Accumulation Mechanism of Pediococcus acidilactici and Weissella confusa in Soy Sauce and the Effects of Phenolic Compound on Citrulline Accumulation
Source: Front Microbiol. 2021 Dec 3;12:757542. doi: 10.3389/fmicb.2021.757542 (PMC8678507; doi:10.3389/fmicb.2021.757542)
Supplement: Supplementary file 1 [file Data_Sheet_1.pdf]

Supplementary Material:

Citrulline accumulation mechanism of *Pediococcus acidilactici* and *Weissella confusa* in soy sauce and the effects of phenolic compound on citrulline accumulation

Kai Zhou [1,2,3], Xiao Zhang [1], Bingyong Li [1], Chaoqun Shen[3], Yuan-Ming Sun[2], Jianyuan Yang [1], Zhen-Lin Xu[2,]*

1 Institute of Jiangxi Oil-tea Camellia, Jiujiang University, Jiujiang, 332000, China

2 Guangdong Provincial Key Laboratory of Food Quality and Safety, College of Food Science, South China Agricultural University, Guangzhou 510642, China

3 Department of production-learning-research, Shenzhen Total-Test Technology Co., td, Shenzhen 518100, China

*Corresponding Author: Zhen-Lin Xu

E-mail: jallent@163.com,

Table S1 The parameters of central composite experimental design experiment

**factor**	**unit**	**low**	**High**	**+alpha**	**-alpha**
pH		4.8	6	4.2	6.6
temperature	°C	15	37	4	48
quercetin	mg/L	10	100	0	145
Gallic acid	mg/L	10	100	0	145

Table S2 Primers used for RT-qPCR analysis of target and reference genes of the two key strains

Strain	Gene		Primer 5'-3'	Amplification efficiency	R^2 for standard curve	Reference
**Target gene**						
W. confusa	*arcA*	F	CAYGCNATGATGCAYYTNGAYACNGT	2.05	0.9951	(Araque et al. 2009)
		R	GTRTTNSWNCCRTCRTTCCAYTGYTC			
	arcB	F	ATGCAYTGYYTNCCNGCNTTYCAYGA	1.88	0.9943	
		R	CCNARNGTNGCNGCCATDATNGCYTT			
	arcC	F	CAYGGNAAYGGNCCNCARGTNGGNAA	1.90	0.9975	
		R	CKNCKNYANCCNCKNCCNGCRTCYTC			
P. acidilactici	*arcA*	F	CTCACGACGGATTGATGGAATAC	1.93	0.9965	(Zhang 2016)
		R	GTCACGAGTAAAGTAAGCATTTGG			
	arcB	F	TGCTGCTAAGACTGGTGCTAAG	1.95	0.9930	
		R	GGTATGGCTTCAACAAGTCAATCC			
	arcC	F	CACGCTCAACGATGTTAGGG	1.91	0.9905	
		R	TTACCGAAACTGACGAAGGC			
**Reference gene**						
W. confusa	*16s*	F	CGTGGGAAACCTACCTCT TA	1.89	0.9993	(Jichan et al. 2002)
		R	CCCTCAAACATCTAGCAC			
P. acidilactici	*16s*	F	GTAGCGGTGAAATGCGTAG	1.93	0.9984	(Zhang 2016)
		R	CGGATTACTTAATGCGTTAGC			

Note: Y: T/C, N: A/T/C/G, R: A/G, S: G/C, W: A/T, D: G/A/T, K: G/T

Table S3 Citrulline accumulation ability of *W. confusa* and *P. acidilactici* in modified *moromi* medium cultured for 7 d

**mg/L**		**Arginine**	**citrulline**	**A-C rate**
Original *moromi* broth		1330.2±124.1	570.0±74.3	-
Fortified with10 mg/L arginine	*W. confusa*	8325±172.8	2292.7±202.6	51.3%
	P. acidilactici	7624.0±59.3	2470.9±238.4	44.0%

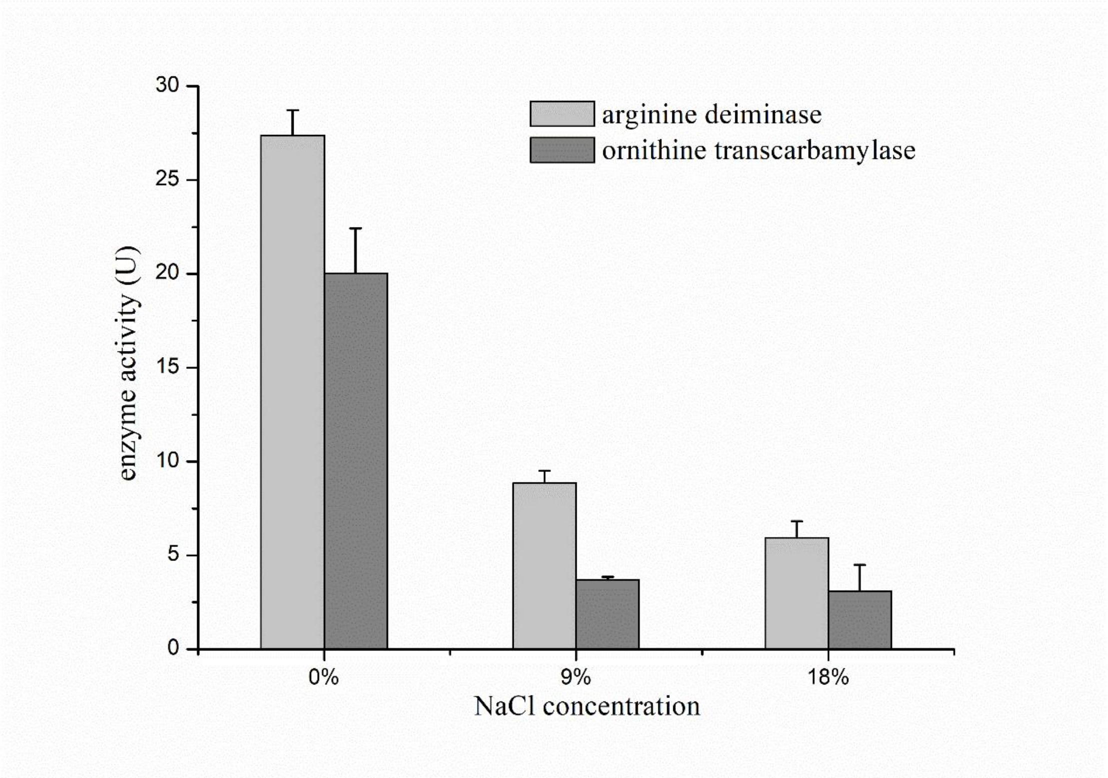

Figure S1 Enzyme activities of ADI and OTC in *P. acidilactici*

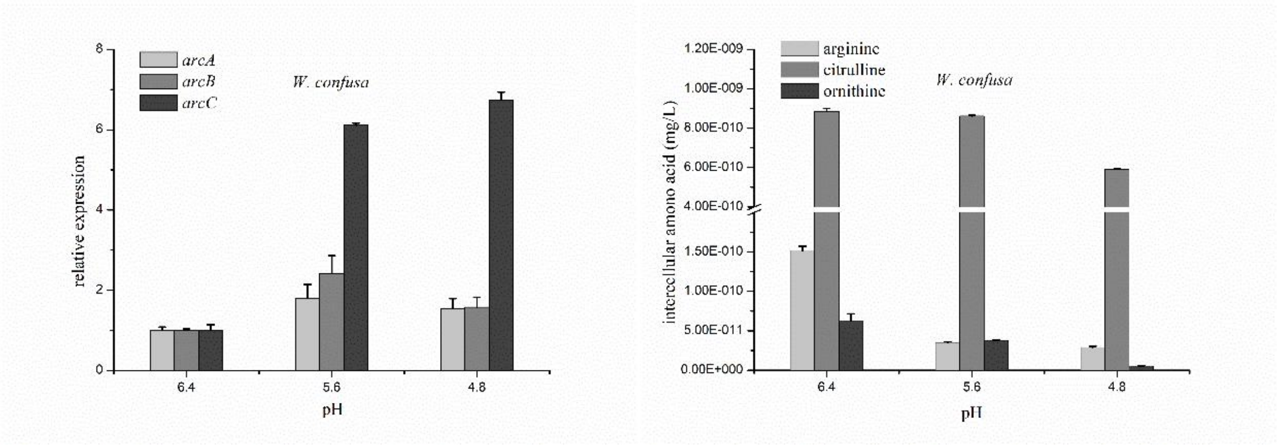

Figure S2 Effect of pH on *arc* gene expression and intracellular amino acid content of *W. confusa* at 18% NaCl

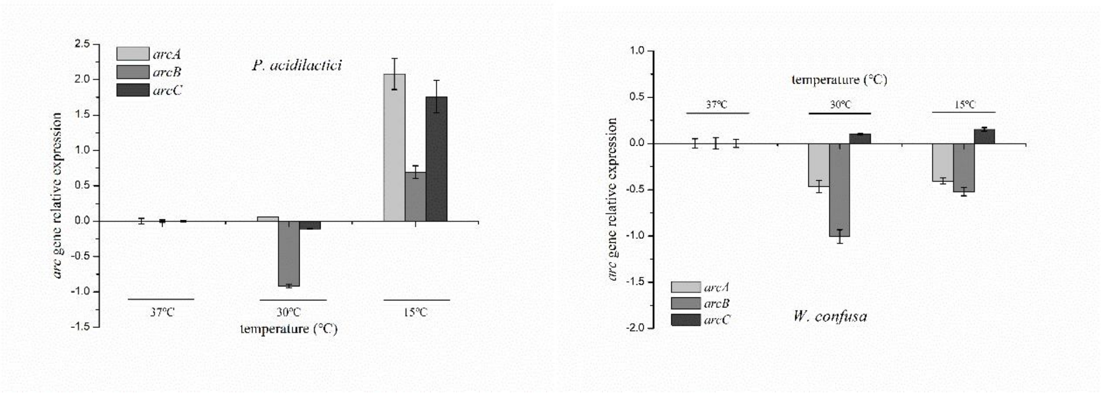

Figure S3 *arc* gene relative expression of *P. acidilactici* and *W. confusa* cultivated in the medium with 18% NaCl at different temperature

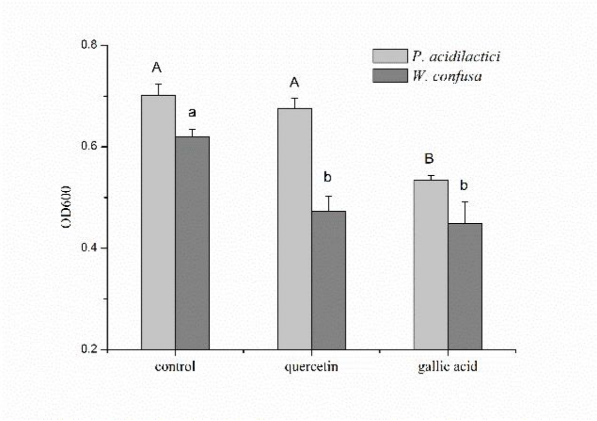

Figure S4 Effect of quercetin and gallic acid on strain growth (Different letters mean significant difference at 5% level of probability)

The 16S rDNA sequence of *Pediococcus acidilactici* (original strain number was S-45-3-1):

TGCAAGTCGAACGAACTTCCGTTAATTGATCAGGACGTGCTTGCACTGAATGAGATTTTA
ACACGAAGTGAGTGGCGGACGGGTGAGTAACACGTGGGTAACCTGCCCAGAAGCAGG
GGATAACACCTGGAAACAGATGCTAATACCGTATAACAGAGAAAACCGCCTGGTTTTCT
TTTAAAAGATGGCTCTGCTATCACTTCTGGATGGACCCGCGGCGCATTAGCTAGTTGGTG
AGGTAACGGCTCACCAAGGCGATGATGCGTAGCCGACCTGAGAGGGTAATCGGCCACAT
TGGGACTGAGACACGGCCCAGACTCCTACGGGAGGCAGCAGTAGGGAATCTTCCACAA
TGGACGCAAGTCTGATGGAGCAACGCCGCGTGAGTGAAGAAGGGTTTCGGCTCGTAAA
GCTCTGTTGTTAAAGAAGAACGTGGGTGAGAGTAACTGTTCACCCAGTGACGGTATTTA
ACCAGAAAGCCACGGCTAACTACGTGCCAGCAGCCGCGGTAATACGTAGGTGGCAAGC
GTTATCCGGATTTATTGGGCGTAAAGCGAGCGCAGGCGGTCTTTTAAGTCTAATGTGAAA
GCCTTCGGCTCAACCGAAGAAGTGCATTGGAAACTGGGAGACTTGAGTGCAGAAGAGG
ACAGTGGAACTCCATGTGTAGCGGTGAAATGCGTAGATATATGGAAGAACACCAGTGGC
GAAGGCGGCTGTCTGGTCTGTAACTGACGCTGAGGCTCGAAAGCATGGGTAGCGAACA
GGATTAGATACCCTGGTAGTCCATGCCGTAAACGATGATTACTAAGTGTTGGAGGGTTTC
CGCCCTTCAGTGCTGCAGCTAACGCATTAAGTAATCCGCCTGGGGAGTACGACCGCAAG
GTTGAAACTCAAAAGAATTGACGGGGGCCCGCACAAGCGGTGGAGCATGTGGTTTAAT
TCGAAGCTACGCGAAGAACCTTACCAGGTCTTGACATCTTCTGCCAACCTAAGAGATTA
GGCGTTCCCTTCGGGGACAGAATGACAGGTGGTGCATGGTTGTCGTCAGCTCGTGTCGT
GAGATGTTGGGTTAAGTCCCGCAACGAGCGCAACCCTTATTACTAGTTGCCAGCATTCA
GTTGGGCACTCTAGTGAGACTGCCGGTGACAAACCGGAGGAAGGTGGGGACGACGTCA
AATCATCATGCCCCTTATGACCTGGGCTACACACGTGCTACAATGGATGGTACAACGAGT
CGCGAAACCGCGAGGTTTAGCTAATCTCTTAAAACCATTCTCAGTTCGGACTGTAGGCT
GCAACTCGCCTACACGAAGTCGGAATCGCTAGTAATCGCGGATCAGCATGCCGCGGTGA
ATACGTTCCCGGGCCTTGTACACACCGCCCGTCACACCATGAGAGTTTGTAACACCCAA
AGCCGGTGGGGTAACCTTTTAGGAGCTAGCCGTCT

The 16S rDNA sequence of *Weissella confusa* (original strain number was S-45-2-5):

TACATGCAGTCGAACGCTTTGTGGTTCAACTGATTTGAAGAGCTTGCTCAGATATGACGA
TGGACATTGCAAAGAGTGGCGAACGGGTGAGTAACACGTGGGAAACCTACCTCTTAGC
AGGGGATAACATTTGGAAACAGATGCTAATACCGTATAACAATGACAACCGCATGGTTGT
TATTTAAAAGATGGTTCTGCTATCACTAAGAGATGGTCCCGCGGTGCATTAGCTAGTTGG
TAAGGTAATGGCTTACCAAGGCGATGATGCATAGCCGAGTTGAGAGACTGATCGGCCAC
AATGGGACTGAGACACGGCCCATACTCCTACGGGAGGCAGCAGTAGGGAATCTTCCACA
ATGGGCGAAAGCCTGATGGAGCAACGCCGCGTGTGTGATGAAGGGTTTCGGCTCGTAA
AACACTGTTGTAAGAGAAGAATGACATTGAGAGTAACTGTTCAATGTGTGACGGTATCT
TACCAGAAAGGAACGGCTAAATACGTGCCAGCAGCCGCGGTAATACGTATGTTCCAAGC
GTTATCCGGATTTATTGGGCGTAAAGCGAGCGCAGACGGTTATTTAAGTCTGAAGTGAAA
GCCCTCAGCTCAACTGAGGAATTGCTTTGGAAACTGGATGACTTGAGTGCAGTAGAGGA
AAGTGGAACTCCATGTGTAGCGGTGAAATGCGTAGATATATGGAAGAACACCAGTGGCG
AAGGCGGCTTTCTGGACTGTAACTGACGTTGAGGCTCGAAAGTGTGGGTAGCAAACAG
GATTAGATACCCTGGTAGTCCACACCGTAAACGATGAGTGCTAGGTGTTTGAGGGTTTCC
GCCCTTAAGTGCCGCAGCTAACGCATTAAGCACTCCGCCTGGGGAGTACGACCGCAAGG
TTGAAACTCAAAGGAATTGACGGGGACCCGCACAAGCGGTGGAGCATGTGGTTTAATTC
GAAGCAACGCGAAGAACCTTACCAGGTCTTGACATCCCTTGACAACTCCAGAGATGGA
GCGTTCCCTTCGGGGACAAGGTGACAGGTGGTGCATGGTTGTCGTCAGCTCGTGTCGTG
AGATGTTGGGTTAAGTCCCGCAACGAGCGCAACCCTTATTACTAGTTGCCAGCATTCAGT
TGGGCACTCTAGTGAGACTGCCGGTGACAAACCGGAGGAAGGTGGGGATGACGTCAAA
TCATCATGCCCCTTATGACCTGGGCTACACACGTGCTACAATGGCGTATACAACGAGTTG
CCAACCCGCGAGGGTGAGCTAATCTCTTAAAGTACGTCTCAGTTCGGATTGTAGGCTGC
AACTCGCCTACATGAAGTCGGAATCGCTAGTAATCGCGGATCAGCACGCCGCGGTGAAT
ACGTTCCCGGGTCTTGTACACACCGCCCGTCACACCATGAGAGTTTGTAACACCCAAAG
CCGGTGGGGTAACCTTCGGGAGCCAGCCGTC

The result of 16S rDNA sequence analysis using the blast method:

Sequences producing significant alignments Download New Select columns Show 100

select all *100 sequences selected* GenBank Graphics Distance tree of results New MSA Viewer

Description	Scientific Name	Max Score	Total Score	Query Cover	E value	Per. Ident	Acc. Len	Accession
Pediococcus acidilactici strain 5541 16S ribosomal RNA gene, partial sequence	Pediococcus aci...	2673	2673	100%	0.0	100.00%	1481	MT463550.1
Pediococcus acidilactici strain DHR013 16S ribosomal RNA gene, partial sequence	Pediococcus aci...	2673	2673	100%	0.0	100.00%	1509	MT012259.1
Pediococcus acidilactici strain CACC 537 chromosome, complete genome	Pediococcus aci...	2673	13310	100%	0.0	100.00%	2035984	CP048019.1
Pediococcus acidilactici strain CACC 537 16S ribosomal RNA gene, partial sequence	Pediococcus aci...	2673	2673	100%	0.0	100.00%	1499	MN918113.1
Pediococcus acidilactici strain FCP-1 16S ribosomal RNA gene, partial sequence	Pediococcus aci...	2673	2673	100%	0.0	100.00%	1484	MN367973.1
Pediococcus acidilactici strain ATCC 8042 chromosome, complete genome	Pediococcus aci...	2673	13277	100%	0.0	100.00%	2009598	CP033438.1
Pediococcus acidilactici strain SRCM103444 chromosome, complete genome	Pediococcus aci...	2673	13366	100%	0.0	100.00%	1970727	CP035266.1
Pediococcus acidilactici strain PB22 chromosome, complete genome	Pediococcus aci...	2673	13305	100%	0.0	100.00%	1955616	CP025471.1
Pediococcus acidilactici strain NRCC1 16S ribosomal RNA gene, partial sequence	Pediococcus aci...	2673	2673	100%	0.0	100.00%	1569	KU504251.1

Sequences producing significant alignments Download New Select columns Show 100

select all *100 sequences selected* GenBank Graphics Distance tree of results New MSA Viewer

Description	Scientific Name	Max Score	Total Score	Query Cover	E value	Per. Ident	Acc. Len	Accession
Weissella confusa strain 3172 16S ribosomal RNA gene, partial sequence	Weissella confusa	2676	2676	100%	0.0	100.00%	1479	MT613537.1
Weissella confusa strain 2992 16S ribosomal RNA gene, partial sequence	Weissella confusa	2676	2676	100%	0.0	100.00%	1475	MT611924.1
Weissella confusa strain 2990 16S ribosomal RNA gene, partial sequence	Weissella confusa	2676	2676	100%	0.0	100.00%	1479	MT611922.1
Weissella confusa strain 2879 16S ribosomal RNA gene, partial sequence	Weissella confusa	2676	2676	100%	0.0	100.00%	1480	MT611841.1
Weissella confusa strain 2732 16S ribosomal RNA gene, partial sequence	Weissella confusa	2676	2676	100%	0.0	100.00%	1473	MT611743.1
Weissella confusa strain 2693 16S ribosomal RNA gene, partial sequence	Weissella confusa	2676	2676	100%	0.0	100.00%	1466	MT611710.1
Weissella confusa strain 2547 16S ribosomal RNA gene, partial sequence	Weissella confusa	2676	2676	100%	0.0	100.00%	1474	MT611579.1
Weissella confusa strain 2333 16S ribosomal RNA gene, partial sequence	Weissella confusa	2676	2676	100%	0.0	100.00%	1482	MT604791.1
Weissella confusa strain 1413 16S ribosomal RNA gene, partial sequence	Weissella confusa	2676	2676	100%	0.0	100.00%	1483	MT573822.1

References:

Araque I, Gil J, Carrete R, Bordons A, Reguant C. 2009. Detection of arc genes related with the ethyl carbamate precursors in wine lactic acid bacteria. Journal of Agricultural & Food Chemistry.57:1841-1847.

Jichan J, Bongjoon K, Jongho L, Jeongho K, Gajin J, Han H. 2002. Identification of Weissella species by the genus-specific amplified ribosomal DNA restriction analysis. Fems Microbiology Letters.212:29-34.

Zhang J R. 2016. Formation mechanism and elimination strategy of ethyl carbamate in soy sauce. Jiangnan university.
